# *cis*-Dichlorido­bis(trimethoxy­phos­phine)palladium(II) at 125 K

**DOI:** 10.1107/S1600536809041919

**Published:** 2009-10-17

**Authors:** Alexandra M. Z. Slawin, Paul G. Waddell, J. Derek Woollins

**Affiliations:** a Department of Chemistry, University of St Andrews, St Andrews, KY16 9ST, Scotland

## Abstract

The title compound, [PdCl_2_(C_3_H_9_O_3_P)_2_], which is isotypic with its platinum analogue, adopts a slightly distorted *cis* square-planar geometry for the Pd centre.

## Related literature

For the platinum analogue, see: Bao *et al.* (1987 ▶). For related platinum complexes, see: Slawin *et al.* (2007*a* ▶,*b* ▶). For *cis*-bis­(triisopropoxyphosphino)platinum dichloride, see: Slawin *et al.* (2009 ▶).
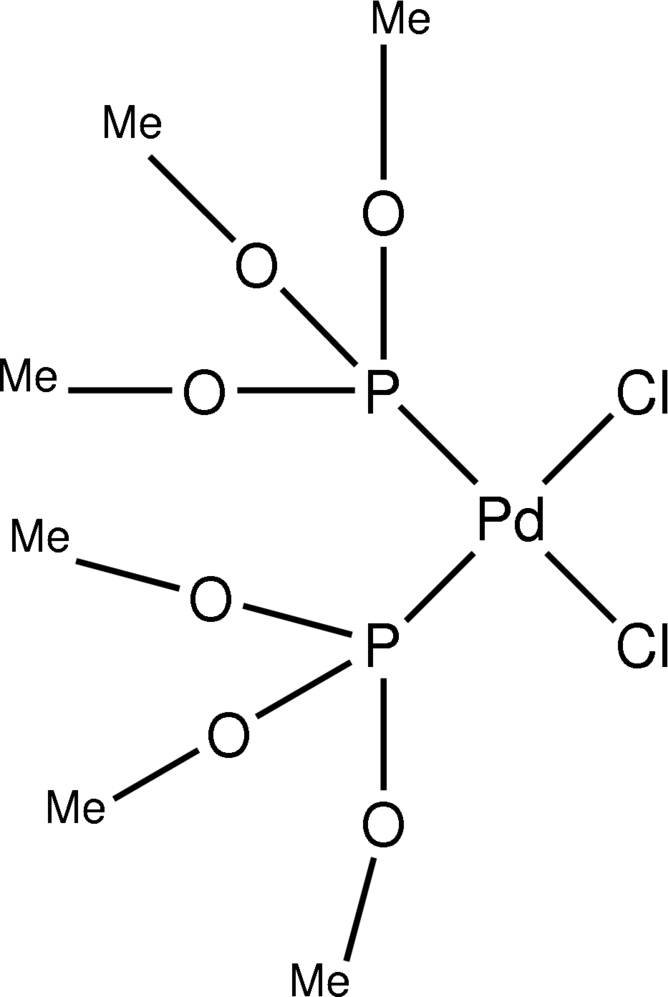


## Experimental

### 

#### Crystal data

[PdCl_2_(C_3_H_9_O_3_P)_2_]
*M* *_r_* = 425.46
Monoclinic, 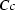

*a* = 6.8059 (19) Å
*b* = 16.897 (5) Å
*c* = 13.374 (4) Å
β = 100.086 (7)°
*V* = 1514.2 (7) Å^3^
*Z* = 4
Mo *K*α radiation
μ = 1.80 mm^−1^
*T* = 125 K
0.22 × 0.16 × 0.13 mm

#### Data collection

Rigaku SCXmini diffractometer
Absorption correction: multi-scan (*ABSCOR*; Higashi, 1995 ▶) *T*_min_ = 0.662, *T*_max_ = 0.791
6333 measured reflections
2639 independent reflections
2338 reflections with *I* > 2σ(*I*)
*R*_int_ = 0.072

#### Refinement

*R*[*F* ^2^ > 2σ(*F* ^2^)] = 0.054
*wR*(*F* ^2^) = 0.090
*S* = 1.07
2639 reflections
161 parameters
H-atom parameters constrained
Δρ_max_ = 1.26 e Å^−3^
Δρ_min_ = −0.71 e Å^−3^
Absolute structure: Flack (1983 ▶), 1308 Friedel pairs
Flack parameter: −0.01 (5)

### 

Data collection: *SCXmini* (Rigaku, 2006 ▶); cell refinement: *PROCESS-AUTO* (Rigaku, 1998 ▶); data reduction: *PROCESS-AUTO*; program(s) used to solve structure: *SHELXS97* (Sheldrick, 2008 ▶); program(s) used to refine structure: *SHELXL97* (Sheldrick, 2008 ▶); molecular graphics: *CrystalStructure* (Rigaku, 2006 ▶); software used to prepare material for publication: *CrystalStructure*.

## Supplementary Material

Crystal structure: contains datablocks General, I. DOI: 10.1107/S1600536809041919/fi2085sup1.cif

Structure factors: contains datablocks I. DOI: 10.1107/S1600536809041919/fi2085Isup2.hkl

Additional supplementary materials:  crystallographic information; 3D view; checkCIF report

## Figures and Tables

**Table:** 

Pd1—Cl1	2.356 (2)
Pd1—Cl2	2.358 (2)
Pd1—P1	2.241 (2)
Pd1—P2	2.233 (2)

**Table:** 

Cl1—Pd1—Cl2	89.71 (8)
Cl1—Pd1—P1	179.04 (9)
Cl1—Pd1—P2	87.59 (8)
Cl2—Pd1—P1	90.32 (8)
Cl2—Pd1—P2	177.30 (8)
P1—Pd1—P2	92.38 (8)

**Fig. 1.**
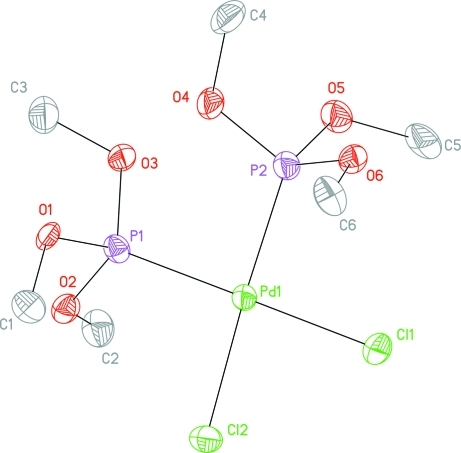
The structure of the title compound with displacement ellipsoids drawn at the 50% probability level, hydrogen atoms omitted for clarity.

## supplementary crystallographic information

### Comment

[PdCl_2_(P(OMe)_3_)_2_] is isomorphous with its platinum analogue [PtCl_2_(P(OMe)_3_)_2_] (2) (Bao *et al.*, 1987) and adopts a *cis* square planar geometry, Whereas the Pt complex was reported to have quite dissmilar bond lengths for Pt—P1/Pt—P2 and Pt—Cl1/PtCl2 bond lengths these pairs of bonds are equivalent in the title compound.

The Pd—Cl bond bonds in (1) ( Pd(1)—Cl(1) 2.356?(2), Pd(1)—Cl(2) 2.358?(2) Å ) are shorter than the Pt—Cl bonds in (2) whilst the Pd—P bonds ( Pd(1)—P(1) 2.241?(2), Pd(1)—P(2) 2.233?(2) Å ) are longer than the Pt—P bonds in (2). The P—M—P angle ( P(1)—Pd(1)—P(2) 92.38?(8) ° ) is reduced and the Cl—M—Cl angle is enlarged ( Cl(1)—Pd(1)—Cl(2) 89.71?(8) ° ) on going from Pt to Pd with the palladium compound reported here having angles which are closer to ideal square planar .

### Experimental

0.5 g (1.75 mmol) of PdCl_2_(COD) was dissolved in dichloromethane (5 mL) in a round-bottomed flask. To this 0.41 mL (3.5 mmol) of trimethylphosphite was added. The solution was stirred for 0.5 h at room temperature. The product was precipitated *via* slow diffusion of hexane and was then filtered off and dried under vacuum, [PdCl_2_(P(OMe)_3_)_2_] (1.36 mmol, *ca* 77%). ^31^P-{^1^H}NMR: δ 97.9 p.p.m..

### Refinement

All H atoms were included in calculated positions and refined as riding atoms with Uĩso~(H) = 1.5 U~eq~. The highest peak in the difference map is 1.097 Å from atom Pd1.

### Crystal data

**Table:**

[PdCl_2_(C_3_H_9_O_3_P)_2_]	*F*(000) = 848.00
*M**_r_* = 425.46	*D*_x_ = 1.866 Mg m^−^^3^
Monoclinic, *C**c*	Mo *K*α radiation, λ = 0.71075 Å
Hall symbol: C -2yc	Cell parameters from 7139 reflections
*a* = 6.8059 (19) Å	θ = 3.1–27.7°
*b* = 16.897 (5) Å	µ = 1.80 mm^−^^1^
*c* = 13.374 (4) Å	*T* = 125 K
β = 100.086 (7)°	Chunk, yellow
*V* = 1514.2 (7) Å^3^	0.22 × 0.16 × 0.13 mm
*Z* = 4	

### Data collection

**Table:**

Rigaku SCXmini diffractometer	2639 independent reflections
Radiation source: fine-focus sealed tube	2338 reflections with *F*^2^ > 2.0σ(*F*^2^)
graphite	*R*_int_ = 0.072
Detector resolution: 6.85 pixels mm^-1^	θ_max_ = 25.4°, θ_min_ = 2.1°
ω scans	*h* = −8→8
Absorption correction: multi-scan (*ABSCOR*; Higashi, 1995)	*k* = −20→20
*T*_min_ = 0.662, *T*_max_ = 0.791	*l* = −16→16
6333 measured reflections	

### Refinement

**Table:**

Refinement on *F*^2^	H-atom parameters constrained
*R*[*F*^2^ > 2σ(*F*^2^)] = 0.054	*w* = 1/[σ^2^(*F*_o_^2^) + (0.0247*P*)^2^] where *P* = (*F*_o_^2^ + 2*F*_c_^2^)/3
*wR*(*F*^2^) = 0.090	(Δ/σ)_max_ < 0.001
*S* = 1.07	Δρ_max_ = 1.26 e Å^−^^3^
2639 reflections	Δρ_min_ = −0.71 e Å^−^^3^
161 parameters	Absolute structure: Flack,(1983), 1308 Friedel pairs
Primary atom site location: structure-invariant direct methods	Flack parameter: −0.01 (5)
Hydrogen site location: inferred from neighbouring sites	

### Special details

**Table:**

Geometry. ENTER SPECIAL DETAILS OF THE MOLECULAR GEOMETRY
Refinement. Refinement was performed using all reflections. The weighted *R*-factor (*wR*) and goodness of fit (*S*) are based on *F*^2^. *R*-factor (gt) are based on *F*. The threshold expression of *F*^2^ > 2.0 σ(*F*^2^) is used only for calculating *R*-factor (gt).

### Fractional atomic coordinates and isotropic or equivalent isotropic displacement parameters (Å^2^)

**Table:**

	*x*	*y*	*z*	*U*_iso_*/*U*_eq_	
Pd(1)	0.50973 (10)	0.10743 (4)	0.45788 (7)	0.01777 (16)	
Cl(1)	0.4602 (3)	0.23766 (14)	0.39555 (17)	0.0298 (6)	
Cl(2)	0.2407 (3)	0.06430 (14)	0.33413 (17)	0.0277 (6)	
P(1)	0.5605 (3)	−0.01677 (14)	0.51540 (16)	0.0183 (5)	
P(2)	0.7614 (3)	0.15409 (14)	0.57243 (17)	0.0201 (5)	
O(1)	0.7314 (8)	−0.0612 (3)	0.4734 (4)	0.0215 (14)	
O(2)	0.3841 (8)	−0.0766 (3)	0.4858 (4)	0.0210 (14)	
O(3)	0.6237 (9)	−0.0227 (3)	0.6348 (4)	0.0252 (15)	
O(4)	0.9294 (8)	0.0917 (3)	0.6089 (4)	0.0259 (15)	
O(5)	0.6928 (8)	0.1816 (3)	0.6740 (4)	0.0267 (15)	
O(6)	0.8710 (8)	0.2305 (3)	0.5430 (4)	0.0202 (14)	
C(1)	0.7285 (16)	−0.0650 (6)	0.3643 (6)	0.034 (2)	
C(2)	0.1966 (14)	−0.0676 (5)	0.5230 (7)	0.030 (2)	
C(3)	0.6711 (15)	−0.0993 (5)	0.6843 (7)	0.033 (2)	
C(4)	1.1106 (14)	0.1149 (5)	0.6777 (7)	0.036 (2)	
C(5)	0.5731 (15)	0.2526 (5)	0.6788 (6)	0.032 (2)	
C(6)	0.9618 (13)	0.2290 (5)	0.4527 (8)	0.032 (2)	
H(1)	0.7662	−0.1183	0.3459	0.041*	
H(2)	0.8233	−0.0265	0.3455	0.041*	
H(3)	0.5939	−0.0527	0.3281	0.041*	
H(4)	0.2211	−0.0723	0.5972	0.037*	
H(5)	0.1031	−0.1090	0.4935	0.037*	
H(6)	0.1394	−0.0155	0.5034	0.037*	
H(7)	0.6000	−0.1045	0.7417	0.040*	
H(8)	0.8152	−0.1028	0.7089	0.040*	
H(9)	0.6298	−0.1419	0.6354	0.040*	
H(10)	1.2047	0.1387	0.6388	0.043*	
H(11)	1.1716	0.0681	0.7138	0.043*	
H(12)	1.0774	0.1534	0.7269	0.043*	
H(13)	0.6614	0.2980	0.6975	0.038*	
H(14)	0.4874	0.2454	0.7298	0.038*	
H(15)	0.4901	0.2621	0.6123	0.038*	
H(16)	1.0930	0.2546	0.4672	0.038*	
H(17)	0.8764	0.2575	0.3977	0.038*	
H(18)	0.9775	0.1740	0.4321	0.038*	

### Atomic displacement parameters (Å^2^)

**Table:**

	*U*^11^	*U*^22^	*U*^33^	*U*^12^	*U*^13^	*U*^23^
Pd(1)	0.0217 (3)	0.0152 (3)	0.0159 (3)	0.0006 (3)	0.0018 (2)	−0.0001 (3)
Cl(1)	0.0375 (15)	0.0183 (13)	0.0301 (14)	0.0010 (12)	−0.0040 (11)	0.0048 (10)
Cl(2)	0.0321 (14)	0.0253 (14)	0.0223 (13)	−0.0027 (12)	−0.0050 (11)	0.0006 (10)
P(1)	0.0228 (14)	0.0171 (13)	0.0157 (13)	−0.0017 (11)	0.0056 (10)	−0.0012 (9)
P(2)	0.0234 (15)	0.0173 (14)	0.0193 (13)	−0.0010 (11)	0.0031 (11)	−0.0002 (10)
O(1)	0.031 (3)	0.019 (3)	0.014 (3)	0.013 (3)	0.003 (2)	0.002 (2)
O(2)	0.024 (3)	0.019 (3)	0.020 (3)	0.001 (2)	0.004 (2)	0.004 (2)
O(3)	0.038 (4)	0.019 (3)	0.016 (3)	0.005 (3)	−0.002 (2)	0.002 (2)
O(4)	0.021 (3)	0.017 (3)	0.037 (3)	0.000 (2)	−0.003 (3)	−0.001 (2)
O(5)	0.036 (4)	0.026 (3)	0.019 (3)	0.000 (3)	0.006 (2)	−0.002 (2)
O(6)	0.025 (3)	0.019 (3)	0.017 (3)	0.000 (2)	0.005 (2)	0.004 (2)
C(1)	0.050 (7)	0.038 (6)	0.014 (5)	0.015 (5)	0.006 (5)	−0.010 (4)
C(2)	0.030 (6)	0.026 (5)	0.036 (6)	−0.002 (5)	0.008 (4)	−0.002 (5)
C(3)	0.046 (6)	0.024 (5)	0.030 (5)	0.006 (5)	0.006 (4)	−0.002 (4)
C(4)	0.026 (5)	0.029 (6)	0.047 (6)	0.006 (4)	−0.007 (4)	0.007 (5)
C(5)	0.045 (6)	0.036 (6)	0.016 (5)	−0.003 (5)	0.011 (4)	−0.009 (4)
C(6)	0.037 (8)	0.028 (5)	0.033 (5)	−0.005 (4)	0.013 (5)	−0.008 (5)

### Geometric parameters (Å, °)

**Table:**

Pd(1)—Cl(1)	2.356 (2)	C(1)—H(2)	0.980
Pd(1)—Cl(2)	2.358 (2)	C(1)—H(3)	0.980
Pd(1)—P(1)	2.241 (2)	C(2)—H(4)	0.980
Pd(1)—P(2)	2.233 (2)	C(2)—H(5)	0.980
P(1)—O(1)	1.568 (6)	C(2)—H(6)	0.980
P(1)—O(2)	1.567 (6)	C(3)—H(7)	0.980
P(1)—O(3)	1.583 (5)	C(3)—H(8)	0.980
P(2)—O(4)	1.568 (6)	C(3)—H(9)	0.980
P(2)—O(5)	1.582 (6)	C(4)—H(10)	0.980
P(2)—O(6)	1.575 (6)	C(4)—H(11)	0.980
O(1)—C(1)	1.457 (10)	C(4)—H(12)	0.980
O(2)—C(2)	1.457 (12)	C(5)—H(13)	0.980
O(3)—C(3)	1.464 (10)	C(5)—H(14)	0.980
O(4)—C(4)	1.458 (10)	C(5)—H(15)	0.980
O(5)—C(5)	1.458 (11)	C(6)—H(16)	0.980
O(6)—C(6)	1.451 (12)	C(6)—H(17)	0.980
C(1)—H(1)	0.980	C(6)—H(18)	0.980
			
Cl(1)···O(5)^i^	3.473 (5)	H(4)···O(6)^iii^	3.594
Cl(1)···C(5)^i^	3.563 (8)	H(4)···C(4)^viii^	3.466
O(1)···C(2)^ii^	3.121 (11)	H(4)···C(5)^iii^	3.367
O(2)···O(6)^iii^	3.352 (7)	H(4)···H(8)^viii^	3.402
O(2)···C(6)^iii^	3.367 (10)	H(4)···H(11)^viii^	2.891
O(3)···C(1)^iv^	3.369 (10)	H(4)···H(13)^iii^	2.636
O(4)···C(2)^ii^	3.550 (11)	H(4)···H(15)^iii^	3.232
O(5)···Cl(1)^v^	3.473 (5)	H(5)···Cl(1)^iii^	2.989
O(5)···C(1)^iv^	3.194 (11)	H(5)···O(1)^viii^	2.624
O(6)···O(2)^vi^	3.352 (7)	H(5)···O(6)^iii^	3.271
C(1)···O(3)^vii^	3.369 (10)	H(5)···C(1)^viii^	2.915
C(1)···O(5)^vii^	3.194 (11)	H(5)···C(5)^iii^	3.440
C(1)···C(2)^ii^	3.506 (13)	H(5)···H(1)^viii^	2.756
C(2)···O(1)^viii^	3.121 (11)	H(5)···H(2)^viii^	2.858
C(2)···O(4)^viii^	3.550 (11)	H(5)···H(13)^iii^	3.113
C(2)···C(1)^viii^	3.506 (13)	H(5)···H(15)^iii^	2.879
C(5)···Cl(1)^v^	3.563 (8)	H(5)···H(17)^iii^	3.321
C(6)···O(2)^vi^	3.367 (10)	H(6)···O(1)^viii^	2.842
Pd(1)···H(7)^vii^	3.060	H(6)···O(4)^viii^	2.832
Pd(1)···H(10)^viii^	3.493	H(6)···C(1)^viii^	3.188
Cl(1)···H(1)^ix^	2.793	H(6)···C(4)^viii^	3.238
Cl(1)···H(5)^vi^	2.989	H(6)···H(1)^viii^	3.469
Cl(1)···H(7)^vii^	3.300	H(6)···H(2)^viii^	2.745
Cl(1)···H(12)^i^	3.121	H(6)···H(10)^viii^	3.158
Cl(1)···H(13)^i^	3.103	H(6)···H(11)^viii^	3.121
Cl(1)···H(14)^i^	3.585	H(6)···H(18)^viii^	3.466
Cl(1)···H(16)^viii^	2.843	H(7)···Pd(1)^iv^	3.060
Cl(1)···H(18)^viii^	3.571	H(7)···Cl(1)^iv^	3.300
Cl(2)···H(2)^viii^	3.255	H(7)···Cl(2)^iv^	3.005
Cl(2)···H(4)^vii^	3.151	H(7)···C(1)^iv^	3.339
Cl(2)···H(7)^vii^	3.005	H(7)···H(2)^iv^	2.897
Cl(2)···H(8)^x^	3.149	H(7)···H(3)^iv^	2.900
Cl(2)···H(11)^x^	2.747	H(7)···H(13)^iii^	3.370
Cl(2)···H(13)^i^	2.949	H(7)···H(18)^iv^	3.489
Cl(2)···H(18)^viii^	3.031	H(8)···Cl(2)^xiv^	3.149
O(1)···H(4)^ii^	3.457	H(8)···C(5)^xi^	3.076
O(1)···H(5)^ii^	2.624	H(8)···H(2)^iv^	2.842
O(1)···H(6)^ii^	2.842	H(8)···H(3)^iv^	3.543
O(1)···H(16)^iii^	3.250	H(8)···H(4)^ii^	3.402
O(2)···H(16)^iii^	3.216	H(8)···H(13)^xi^	2.917
O(2)···H(17)^iii^	3.038	H(8)···H(14)^xi^	2.814
O(3)···H(2)^iv^	3.022	H(8)···H(15)^xi^	2.973
O(3)···H(3)^iv^	2.920	H(8)···H(18)^iv^	3.229
O(4)···H(1)^iv^	3.567	H(9)···O(6)^iii^	2.917
O(4)···H(2)^iv^	3.543	H(9)···C(5)^xi^	3.465
O(4)···H(4)^ii^	3.429	H(9)···C(6)^iii^	3.325
O(4)···H(6)^ii^	2.832	H(9)···H(13)^iii^	3.578
O(5)···H(1)^iv^	2.506	H(9)···H(14)^xi^	3.173
O(5)···H(2)^iv^	3.494	H(9)···H(15)^xi^	3.001
O(5)···H(3)^iv^	3.151	H(9)···H(16)^iii^	2.826
O(5)···H(10)^viii^	3.352	H(10)···Pd(1)^ii^	3.493
O(6)···H(4)^vi^	3.594	H(10)···O(5)^ii^	3.352
O(6)···H(5)^vi^	3.271	H(10)···C(5)^ii^	3.132
O(6)···H(9)^vi^	2.917	H(10)···H(6)^ii^	3.158
C(1)···H(5)^ii^	2.915	H(10)···H(14)^ii^	2.759
C(1)···H(6)^ii^	3.188	H(10)···H(15)^ii^	2.914
C(1)···H(7)^vii^	3.339	H(11)···Cl(2)^xiv^	2.747
C(1)···H(12)^vii^	3.575	H(11)···C(2)^ii^	3.457
C(1)···H(16)^iii^	3.534	H(11)···H(2)^iv^	3.268
C(2)···H(1)^viii^	3.535	H(11)···H(3)^xiv^	3.021
C(2)···H(2)^viii^	3.235	H(11)···H(4)^ii^	2.891
C(2)···H(11)^viii^	3.457	H(11)···H(6)^ii^	3.121
C(2)···H(13)^iii^	3.293	H(12)···Cl(1)^v^	3.121
C(2)···H(15)^iii^	3.502	H(12)···C(1)^iv^	3.575
C(3)···H(2)^iv^	3.075	H(12)···H(1)^iv^	2.925
C(3)···H(3)^iv^	3.305	H(12)···H(2)^iv^	3.327
C(3)···H(14)^xi^	3.381	H(12)···H(14)^ii^	3.188
C(3)···H(15)^xi^	3.443	H(12)···H(17)^v^	3.161
C(4)···H(1)^iv^	3.522	H(13)···Cl(1)^v^	3.103
C(4)···H(2)^iv^	3.554	H(13)···Cl(2)^v^	2.949
C(4)···H(4)^ii^	3.466	H(13)···C(2)^vi^	3.293
C(4)···H(6)^ii^	3.238	H(13)···H(4)^vi^	2.636
C(4)···H(14)^ii^	3.361	H(13)···H(5)^vi^	3.113
C(5)···H(1)^iv^	3.293	H(13)···H(7)^vi^	3.370
C(5)···H(4)^vi^	3.367	H(13)···H(8)^ix^	2.917
C(5)···H(5)^vi^	3.440	H(13)···H(9)^vi^	3.578
C(5)···H(8)^ix^	3.076	H(14)···Cl(1)^v^	3.585
C(5)···H(9)^ix^	3.465	H(14)···C(3)^ix^	3.381
C(5)···H(10)^viii^	3.132	H(14)···C(4)^viii^	3.361
C(5)···H(17)^xii^	3.430	H(14)···C(6)^xii^	3.047
C(6)···H(9)^vi^	3.325	H(14)···H(1)^iv^	3.097
C(6)···H(14)^xiii^	3.047	H(14)···H(3)^iv^	3.538
H(1)···Cl(1)^xi^	2.793	H(14)···H(8)^ix^	2.814
H(1)···O(4)^vii^	3.567	H(14)···H(9)^ix^	3.173
H(1)···O(5)^vii^	2.506	H(14)···H(10)^viii^	2.759
H(1)···C(2)^ii^	3.535	H(14)···H(12)^viii^	3.188
H(1)···C(4)^vii^	3.522	H(14)···H(16)^xii^	3.131
H(1)···C(5)^vii^	3.293	H(14)···H(17)^xii^	2.492
H(1)···H(5)^ii^	2.756	H(14)···H(18)^xii^	3.040
H(1)···H(6)^ii^	3.469	H(15)···C(2)^vi^	3.502
H(1)···H(12)^vii^	2.925	H(15)···C(3)^ix^	3.443
H(1)···H(14)^vii^	3.097	H(15)···H(4)^vi^	3.232
H(1)···H(16)^iii^	3.050	H(15)···H(5)^vi^	2.879
H(1)···H(17)^iii^	3.545	H(15)···H(8)^ix^	2.973
H(2)···Cl(2)^ii^	3.255	H(15)···H(9)^ix^	3.001
H(2)···O(3)^vii^	3.022	H(15)···H(10)^viii^	2.914
H(2)···O(4)^vii^	3.543	H(15)···H(16)^viii^	3.041
H(2)···O(5)^vii^	3.494	H(16)···Cl(1)^ii^	2.843
H(2)···C(2)^ii^	3.235	H(16)···O(1)^vi^	3.250
H(2)···C(3)^vii^	3.075	H(16)···O(2)^vi^	3.216
H(2)···C(4)^vii^	3.554	H(16)···C(1)^vi^	3.534
H(2)···H(5)^ii^	2.858	H(16)···H(1)^vi^	3.050
H(2)···H(6)^ii^	2.745	H(16)···H(9)^vi^	2.826
H(2)···H(7)^vii^	2.897	H(16)···H(14)^xiii^	3.131
H(2)···H(8)^vii^	2.842	H(16)···H(15)^ii^	3.041
H(2)···H(11)^vii^	3.268	H(17)···O(2)^vi^	3.038
H(2)···H(12)^vii^	3.327	H(17)···C(5)^xiii^	3.430
H(3)···O(3)^vii^	2.920	H(17)···H(1)^vi^	3.545
H(3)···O(5)^vii^	3.151	H(17)···H(5)^vi^	3.321
H(3)···C(3)^vii^	3.305	H(17)···H(12)^i^	3.161
H(3)···H(7)^vii^	2.900	H(17)···H(14)^xiii^	2.492
H(3)···H(8)^vii^	3.543	H(18)···Cl(1)^ii^	3.571
H(3)···H(11)^x^	3.021	H(18)···Cl(2)^ii^	3.031
H(3)···H(14)^vii^	3.538	H(18)···H(6)^ii^	3.466
H(4)···Cl(2)^iv^	3.151	H(18)···H(7)^vii^	3.489
H(4)···O(1)^viii^	3.457	H(18)···H(8)^vii^	3.229
H(4)···O(4)^viii^	3.429	H(18)···H(14)^xiii^	3.040
			
Cl(1)—Pd(1)—Cl(2)	89.71 (8)	O(2)—C(2)—H(4)	109.5
Cl(1)—Pd(1)—P(1)	179.04 (9)	O(2)—C(2)—H(5)	109.5
Cl(1)—Pd(1)—P(2)	87.59 (8)	O(2)—C(2)—H(6)	109.5
Cl(2)—Pd(1)—P(1)	90.32 (8)	H(4)—C(2)—H(5)	109.5
Cl(2)—Pd(1)—P(2)	177.30 (8)	H(4)—C(2)—H(6)	109.5
P(1)—Pd(1)—P(2)	92.38 (8)	H(5)—C(2)—H(6)	109.5
Pd(1)—P(1)—O(1)	113.9 (2)	O(3)—C(3)—H(7)	109.5
Pd(1)—P(1)—O(2)	116.9 (2)	O(3)—C(3)—H(8)	109.5
Pd(1)—P(1)—O(3)	113.8 (2)	O(3)—C(3)—H(9)	109.5
O(1)—P(1)—O(2)	100.6 (3)	H(7)—C(3)—H(8)	109.5
O(1)—P(1)—O(3)	104.1 (3)	H(7)—C(3)—H(9)	109.5
O(2)—P(1)—O(3)	106.0 (3)	H(8)—C(3)—H(9)	109.5
Pd(1)—P(2)—O(4)	113.9 (2)	O(4)—C(4)—H(10)	109.5
Pd(1)—P(2)—O(5)	112.8 (2)	O(4)—C(4)—H(11)	109.5
Pd(1)—P(2)—O(6)	117.3 (2)	O(4)—C(4)—H(12)	109.5
O(4)—P(2)—O(5)	103.9 (3)	H(10)—C(4)—H(11)	109.5
O(4)—P(2)—O(6)	106.1 (3)	H(10)—C(4)—H(12)	109.5
O(5)—P(2)—O(6)	101.4 (3)	H(11)—C(4)—H(12)	109.5
P(1)—O(1)—C(1)	119.9 (5)	O(5)—C(5)—H(13)	109.5
P(1)—O(2)—C(2)	121.6 (5)	O(5)—C(5)—H(14)	109.5
P(1)—O(3)—C(3)	120.7 (5)	O(5)—C(5)—H(15)	109.5
P(2)—O(4)—C(4)	120.4 (5)	H(13)—C(5)—H(14)	109.5
P(2)—O(5)—C(5)	122.1 (5)	H(13)—C(5)—H(15)	109.5
P(2)—O(6)—C(6)	118.9 (5)	H(14)—C(5)—H(15)	109.5
O(1)—C(1)—H(1)	109.5	O(6)—C(6)—H(16)	109.5
O(1)—C(1)—H(2)	109.5	O(6)—C(6)—H(17)	109.5
O(1)—C(1)—H(3)	109.5	O(6)—C(6)—H(18)	109.5
H(1)—C(1)—H(2)	109.5	H(16)—C(6)—H(17)	109.5
H(1)—C(1)—H(3)	109.5	H(16)—C(6)—H(18)	109.5
H(2)—C(1)—H(3)	109.5	H(17)—C(6)—H(18)	109.5
			
Cl(1)—Pd(1)—P(2)—O(4)	−151.9 (2)	O(1)—P(1)—O(2)—C(2)	170.3 (5)
Cl(1)—Pd(1)—P(2)—O(5)	90.0 (2)	O(2)—P(1)—O(1)—C(1)	73.0 (6)
Cl(1)—Pd(1)—P(2)—O(6)	−27.2 (2)	O(1)—P(1)—O(3)—C(3)	−52.8 (7)
Cl(2)—Pd(1)—P(1)—O(1)	98.3 (2)	O(3)—P(1)—O(1)—C(1)	−177.4 (6)
Cl(2)—Pd(1)—P(1)—O(2)	−18.5 (2)	O(2)—P(1)—O(3)—C(3)	52.8 (7)
Cl(2)—Pd(1)—P(1)—O(3)	−142.6 (2)	O(3)—P(1)—O(2)—C(2)	62.1 (6)
P(1)—Pd(1)—P(2)—O(4)	27.1 (2)	Pd(1)—P(2)—O(4)—C(4)	175.4 (5)
P(1)—Pd(1)—P(2)—O(5)	−91.0 (2)	Pd(1)—P(2)—O(5)—C(5)	−71.2 (6)
P(1)—Pd(1)—P(2)—O(6)	151.9 (2)	Pd(1)—P(2)—O(6)—C(6)	−58.1 (5)
P(2)—Pd(1)—P(1)—O(1)	−81.8 (2)	O(4)—P(2)—O(5)—C(5)	165.0 (6)
P(2)—Pd(1)—P(1)—O(2)	161.4 (2)	O(5)—P(2)—O(4)—C(4)	−61.6 (7)
P(2)—Pd(1)—P(1)—O(3)	37.3 (2)	O(4)—P(2)—O(6)—C(6)	70.5 (5)
Pd(1)—P(1)—O(1)—C(1)	−52.9 (6)	O(6)—P(2)—O(4)—C(4)	44.8 (7)
Pd(1)—P(1)—O(2)—C(2)	−65.9 (6)	O(5)—P(2)—O(6)—C(6)	178.7 (5)
Pd(1)—P(1)—O(3)—C(3)	−177.3 (5)	O(6)—P(2)—O(5)—C(5)	55.1 (6)

Symmetry codes: (i) *x*−1/2, −*y*+1/2, *z*−1/2; (ii) *x*+1, *y*, *z*; (iii) *x*−1/2, *y*−1/2, *z*; (iv) *x*, −*y*, *z*+1/2; (v) *x*+1/2, −*y*+1/2, *z*+1/2; (vi) *x*+1/2, *y*+1/2, *z*; (vii) *x*, −*y*, *z*−1/2; (viii) *x*−1, *y*, *z*; (ix) *x*−1/2, *y*+1/2, *z*; (x) *x*−1, −*y*, *z*−1/2; (xi) *x*+1/2, *y*−1/2, *z*; (xii) *x*−1/2, −*y*+1/2, *z*+1/2; (xiii) *x*+1/2, −*y*+1/2, *z*−1/2; (xiv) *x*+1, −*y*, *z*+1/2.
